# A Festschrift Celebrating Dr. Dimiter Stanchev Dimitrov: Antibodies, Innovation, and Impact on Infectious Disease and Cancer Research

**DOI:** 10.3390/antib15050078

**Published:** 2026-09-01

**Authors:** Ponraj Prabakaran, Tianlei Ying, Wei Li

**Affiliations:** 1 PMJ Technology Solutions LLC, Frederick, MD 21704, USA; 2 Antibody Engineering and Drug Discovery Group, MOE/MOH Key Laboratory of Medical Molecular Virology, Shanghai Medical College, Fudan University, 131 Dong An Road, Shanghai 200032, China; 3 Division of Infectious Diseases, Department of Medicine, School of Medicine, University of Pittsburgh, Pittsburgh, PA 15213, USA

Antibody therapeutics now span across oncology, infectious disease, and autoimmunity, and the molecules entering development are increasingly being engineered rather than merely selected [1,2]. Full-size immunoglobulins are joined by fragments, engineered domains, bispecific and multispecific formats, antibody–drug conjugates, chimeric antigen receptors, and cell engagers [3,4]. This Special Issue is dedicated to Dr. Dimiter Stanchev Dimitrov, the founding Editor-in-Chief of *Antibodies*, whose work on display and library methodologies and on antibody engineering across these formats has shaped much of this expansion [5,6,7,8,9,10,11,12,13,14]. The contributions collected here reflect this, from the isolation of new binding domains to the arming, characterization, and manufacture of the molecules built from them.

The collection starts with a review. Yang and Massumi survey fragment-based antibodies configured as immune cell engagers for cancer, infectious disease, and autoimmune disease, drawing on the clinical and translational record for these agents (Contribution 1). They trace the clinical outcomes of these agents to a defined set of engineering parameters: persistence, affinity modulation, conditional activation, antigen selectivity, and costimulation of the engaged immune cell.

The single-domain format is addressed from three directions. Chen and colleagues isolated a fully human heavy chain variable domain, VH20, from a phage display library and mapped its epitope to the glycine-rich region of anaplastic lymphoma kinase, with an EC_50_ of 0.4 nM, a K_D_ of 6.54 nM, and no off-target binding across approximately 6000 human membrane proteins (Contribution 2). Bispecific T-cell engager and chimeric antigen receptor T-cell constructs built on VH20 lysed tumor cells in a target-dependent manner and elicited secretions of IL-2, TNFα, and IFNγ. Baselga and colleagues assembled MO-IISA, a curated database of 2053 single-domain antibodies with known targets, drawn from six public resources and four camelid species (Contribution 3). Framework architecture was conserved across species, while CDR2 and CDR3 carried most of the inter-species and intra-species variation, and non-canonical cysteines occurred more frequently in Bactrian and dromedary CDRs. Jalil and colleagues conducted a systematic review of 32 studies published between 2011 and 2025 on nanobody-based immunoassays and biosensing platforms for bacteria and toxins (Contribution 4). The reported platforms reached high sensitivity and thermostability, but nearly all rested on spiked samples rather than on validated clinical or food matrices.

Two articles hold one element of an antibody construct that is fixed and another that varies. Zhou and colleagues built chimeric antigen receptors from an anti-HER2 antibody directed against domain III and from trastuzumab, which binds the juxtamembrane domain IV (Contribution 5). The two antibodies mediated comparable antibody-dependent cellular cytotoxicity, but only the domain IV construct conferred potent antitumor activity on CAR-T cells. Kuhlmann and colleagues conjugated the anti-gp41 antibody 7B2 to deglycosylated ricin A chain, to PNU-159682, and to actinium-225, holding the antibody and its epitope constant and varying only the payload (Contribution 6). The immunotoxin and the radioimmunoconjugate acted more rapidly than the antibody–drug conjugate, and only the antibody–drug conjugate and the radioimmunoconjugate produced bystander killing, the absence of which allowed envelope-negative cells to outgrow under the immunotoxin.

Discrimination between closely related targets is the problem addressed in two articles. Suzuki and colleagues converted the cancer-specific anti-podocalyxin antibody PcMab-60 into a humanized IgG1, humPcMab-60, which bound pancreatic and colorectal cancer lines but not a normal lymphatic endothelial cell line (Contribution 7). The antibody mediated antibody-dependent cellular cytotoxicity against all three cancer lines and reduced xenograft tumor weight by 35 to 48 percent. Silva-Espinoza and colleagues selected a thirteen-residue peptide predicted by AlphaFold modeling and hydropathicity analysis to be surface-exposed on mouse Schlafen 8 and divergent from Schlafen 9 (Contribution 8). Antibodies raised against the conjugated peptide, including hybridoma-derived IgG, distinguished the two proteins, which share 86 percent sequence identity, by Western blot, immunoprecipitation, immunohistochemistry, and ELISA.

Expression and manufacturing are the subject of two articles. Marsili and colleagues compared two EMCV IRES bicistronic vectors differing only in the order of the light- and heavy-chain genes, using two neutralizing anti-SARS-CoV-2 spike IgG1 antibodies as model molecules in transient ExpiCHO culture (Contribution 9). Vectors placing the light chain as the first cistron consistently yielded more antibodies, and circular dichroism, SEC-HPLC, surface plasmon resonance, and glycan profiling showed that quality attributes were retained. Gebhardt and colleagues combined the CHO4Tx transient expression system with in-culture magnetic protein A capture and semi-automated elution and buffer exchange (Contribution 10). The workflow reached titers of 200 mg/L and above and delivered approximately 20 mg of crude material per 100 mL flask, with a throughput of nineteen constructs in a single run.

These contributions span the discovery, engineering, characterization, and production of antibody therapeutics and reagents, and reflect the range of work that Dr. Dimitrov has done much to shape.

## Data Availability

No new data were created or analyzed in this study. Data sharing is not applicable to this article.
